# Micro-Tensile Bond Strength of a Mesoporous Bioactive Glass-Containing Universal Adhesive: An In Vitro Study on the Effects of Artificial Aging

**DOI:** 10.3390/ma18184256

**Published:** 2025-09-11

**Authors:** Jiyoung Kwon, Soyoung Park, Gil-Joo Ryu, Duck-Su Kim

**Affiliations:** 1 Department of Conservative Dentistry, Kyung Hee University Dental Hospital, Seoul 02447, Republic of Korea; jykt55@gmail.com; 2 ONA Dental Clinic, Seoul 05004, Republic of Korea; sy031227@gmail.com; 3 Good Will Dental Hospital, Busan 46547, Republic of Korea; rkjatom@hanmail.net; 4 Department of Conservative Dentistry, School of Dentistry, Kyung Hee University, Seoul 02453, Republic of Korea

**Keywords:** micro-tensile bond strength, mesoporous bioactive glass, universal adhesive, bond durability, resin–dentin interface

## Abstract

**Background:** We evaluated the immediate and artificially aged micro-tensile bond strengths (μTBS) of Hi-Bond Universal, a universal adhesive containing mesoporous bioactive glass (MBG). **Methods:** Human dentin specimens were bonded using the following four application modes: Hi-Bond Universal in etch-and-rinse mode, Hi-Bond Universal in self-etch mode, Single Bond 2 in etch-and-rinse mode, and G-ænial Bond in self-etch mode. Specimens were tested either immediately or after artificial aging (thermocycling or water storage). μTBS values were analyzed statistically, and the resin–dentin interfaces were examined using FE-SEM (Field-emission scanning electron microscopy). **Results:** Results showed that both aging and adhesive mode significantly affected the μTBS (*p* < 0.0001). Immediately after bonding, etch-and-rinse modes produced significantly higher μTBS than the self-etch modes (*p* < 0.0001). Artificial aging reduced bond strength by approximately 30–50%; however, the μTBS of Hi-Bond Universal decreased less than that of Single Bond 2 after water storage. FE-SEM analysis also revealed detachment of the hybrid layer in most adhesives following aging; however, Hi-Bond Universal in the etch-and-rinse mode maintained a relatively intact adhesive layer after water storage. **Conclusion:** Etch-and-rinse application of MBG-containing adhesive may enhance the long-term durability of adhesive restorations.

## 1. Introduction

In contemporary restorative dentistry, adhesive restorations are widely used, largely because of their superior esthetic clinical outcomes and minimally invasive tooth preparation enabled by sophisticated adhesion technologies. The long-term clinical success of such restorations fundamentally depends on establishing and preserving a stable bond between the restorative material and tooth [1]. However, this remains challenging, stemming from factors such as the intrinsic structural and compositional diversity of enamel and dentin, and possible procedural errors during bonding. To overcome these challenges, extensive research has been directed toward refining adhesive formulations, optimizing surface conditioning protocols, and enhancing application methodologies [2]. Such optimization is critical for reinforcing the durability of restorations against mechanical, thermal, and chemical stresses in the oral environment [3,4]. Therefore, the effectiveness of dental adhesives is integral to clinical outcomes, particularly the longevity and reliability of restorations.

Although both etch-and-rinse and self-etch adhesive systems have exhibited favorable immediate bond strengths in previous studies, their long-term durability under various clinical conditions often remains limited [5]. Mechanical stress during occlusion [6,7], volumetric changes at the dentin–adhesive interface due to differences in thermal coefficients [8,9], and degradation of collagen fibers within the hybrid layer may negatively affect adhesive restoration [10,11]. Consequently, significant ongoing research is being dedicated to the development of better dentin adhesives.

The advent of universal adhesives represents a major advancement in adhesive technology. These systems can be applied with etch-and-rinse, self-etch, or selective-etch techniques, allowing clinicians to adapt to various clinical situations. Universal adhesives simplify bonding protocols, reduce procedural complexity, and minimize technique sensitivity, promoting consistent clinical outcomes across diverse restorations [12,13]. A crucial component in most universal adhesives is 10-methacryloyloxydecyl dihydrogen phosphate (10-MDP), a monomer that contributes significantly to adhesive performance through its unique chemical affinity with tooth hydroxyapatite. This monomer forms stable and durable MDP–calcium salts by chemical bonding to the tooth structure, thereby enhancing bond strength and interface stability [14,15]. Moreover, the inclusion of 10-MDP is associated with improved resistance to hydrolytic degradation, further augmenting the longevity and reliability of the adhesive interface in challenging intraoral environments [16,17,18]. The effectiveness of 10-MDP is attributed to its unique structure, which includes a methacrylate group that allows it to polymerize with other resin monomers and a phosphoric acid group that can form strong chemical bonds with calcium in tooth hydroxyapatite. This chemical reaction creates stable nanolayered MDP–calcium salts of MDP–calcium salts on the bonding surface [19]. These layers are highly resistant to breakdown in water or dissolution, which helps maintain both the initial and long-term strength of the bond. Consequently, the hybrid layer remains intact, and there is less leakage at the interface, even under the demanding conditions of the mouth [20]. Furthermore, because 10-MDP can bond well with both enamel and dentin, it can be used with different etching techniques, making it a flexible and valuable component in modern dental adhesives.

Bioactive glass (BAG), first developed by Hench in 1969, is well known for its excellent biocompatibility and ability to form chemical bonds with hard tissues [21]. In physiological environments, BAG undergoes a series of surface reactions, starting with the release of cations and followed by the formation of a silica-rich layer. This layer partially hydrolyzes to form silanol groups, which then repolymerize in a process similar to the mineralization of hard tissues. These reactions gradually convert amorphous calcium phosphate into octacalcium phosphate and finally into hydroxycarbonate apatite, which acts as a nucleation site for hard tissue mineralization [22]. Although BAG is highly bioactive, traditional melt-quenched BAG is too large for use in dental materials. Recent advances in synthesis methods, such as the sol-gel technique, have enabled the production of smaller particles with larger surface areas. Sol-gel-derived BAG created using these advanced methods offers a higher surface area, greater pore volume, and improved apatite-forming ability than conventional BAG, while maintaining good biocompatibility in simulated body fluids [23,24]. Therefore, it overcomes the limitations of traditional BAG and provides superior bioactivity and suitability for dental applications.

According to previous studies, sol-gel-derived BAGs in dentin adhesives demonstrate enhanced remineralization capacity and mechanical properties [25]. In addition, mesoporous bioactive glass (MBG)-containing dental sealants exhibit superior clinical outcomes by improving adhesion and promoting enamel remineralization [26]. A universal adhesive containing MBG has demonstrated dentin hypersensitivity relief potential [27,28]. Recently, Hi-Bond Universal (MEDICLUS, Cheongju, Republic of Korea), a universal dentin adhesive containing MBG, has been introduced. Owing to the unique bioactivity of MBG, its incorporation into dental adhesives may affect bond strength differently than conventional adhesives. Although MBG can maintain the hybrid layer and support long-term tooth health, it may also influence the durability of the adhesive bond over time [29,30]. Therefore, although MBG-containing adhesives may exhibit immediate bond strengths similar to those of traditional adhesives, their bond strengths may decrease with aging.

We aimed to evaluate the long-term durability of an MBG-containing universal adhesive (Hi-Bond Universal) by comparing its bond strength and interface stability with those of conventional adhesives subjected to various artificial aging protocols. The null hypothesis states that there would be no significant difference in the bond strengths under each aging condition of the adhesive with the same application mode.

## 2. Materials and Methods

### 2.1. Study Design

**Figure 1:**
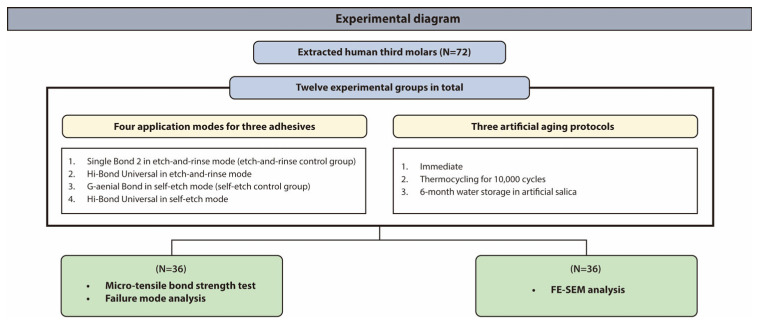
Experimental design of the study. Seventy-two extracted human third molars were allocated to experimental groups according to three aging conditions (immediate, thermocycling, and water storage) and three adhesives (Single Bond 2, G-ænial Bond, and Hi-Bond Universal), each applied in either etch-and-rinse mode or self-etch mode.

The materials and methods used in this study are summarized in Figure 1. Ethical approval was obtained from the Institutional Review Board of Kyung Hee University Dental Hospital (Protocol No. KH-DT22009). According to the protocol, 72 intact, caries-free human third molars were obtained. Following extraction, each tooth was thoroughly cleaned to remove adherent soft tissues and stored at 4 °C in a 0.1% thymol solution to prevent microbial proliferation until further use. Three commercially available dentin adhesive systems were selected: (1) Single Bond 2 (3M ESPE, St. Paul, MN, USA) as an etch-and-rinse control; (2) G-ænial Bond (GC, Tokyo, Japan) as a self-etch control; and (3) Hi-Bond Universal (MEDICLUS). Artificial saliva was prepared for water storage according to Iijima et al. [31]. The compositions of the materials used in this study are listed in Table 1. Figure 1 illustrates the overall design of the experiment.

### 2.2. Micro-Tensile Bond Strength (μTBS) Test

Twelve experimental groups were assigned according to the four application modes of the three adhesives and three artificial aging methods. The sample size for each experimental group was determined based on previous studies using G*Power software (version 3.1.9.7; Heinrich Heine University Düsseldorf, Düsseldorf, Germany) [32,33]. An effective sample size of 20 beams per group was calculated to achieve a statistical power > 0.95 (β = 0.05) with a significance level (α) of 0.01. A total of 36 human third molars were randomly allocated into 12 experimental groups, with three teeth assigned to each group. Each tooth was sectioned to obtain resin–dentin beams for μTBS testing. This allocation ensured standardized distribution across all groups for comparative analysis. The superficial enamel was removed with a high-speed diamond bur, and the exposed occlusal dentin was polished using 600-grit silicon carbide paper under water to produce a uniform smear layer.

For the etch-and-rinse mode, dentin surfaces were conditioned with 35% phosphoric acid gel (Select HV Etch; BISCO, Schaumburg, IL, USA) for 15 s, thoroughly rinsed with water for 30 s using a three-way syringe, and blot-dried. In the self-etch mode, adhesives were applied directly to the prepared dentin surface. Each adhesive was applied according to the manufacturer’s instructions and light-cured using an LED (light-emitting diodes) curing light (Blue Phase 20i; Ivoclar Vivadent, Schaan, Liechtenstein) at 1200 mW/cm^2^ for 20 s. A 4 mm increment of composite resin (Any-Com; MEDICLUS) was then built on the adhesive layer and polymerized under the same conditions.

After composite build-up, each tooth was sectioned longitudinally and transversely using a water-cooled diamond saw (IsoMet 5000; Buehler, Lake Bluff, IL, USA) to obtain composite–dentin beams measuring approximately 1 mm × 1 mm^2^. Approximately 6–12 resin–dentin beams were obtained from each tooth, and the central 6–7 beams per tooth were used for the μTBS testing. Sixty composite–dentin beams from each application mode were randomly divided into three subgroups according to the artificial aging protocol (n = 20).

The artificial aging procedure was performed as follows. First, the specimens from the immediate groups were stored in distilled water at 37 °C for 24 h prior to testing. Second, for the thermocycling groups, the specimens underwent 10,000 thermocycles between water baths maintained at 5 °C and 55 °C (30 s dwell time per bath) using a thermocycler (PJ Tech, Incheon, Republic of Korea) before testing. Third, to simulate long-term intraoral exposure, the specimens were immersed in artificial saliva (Biosesang, Seongnam, Republic of Korea) at 37 °C for 6 months, with the storage medium refreshed every 2 d. After each artificial aging process, a universal testing machine (AGS-X; Shimadzu, Kyoto, Japan) was used to apply a tensile load at a crosshead speed of 1 mm/min until failure, and the maximum load was recorded and converted to stress (MPa) based on the bonded area.

### 2.3. Failure Mode Analysis

For the μTBS measurement, the fracture surfaces of all beams were carefully examined using a stereomicroscope (HD Lite 1080P; Tucsen Photonic, Fuzhou, Fujian, China) at 40× magnification. The observed failure modes were categorized into three categories. Adhesive failures show true interface breakdown of the bond between the restorative material and tooth structure. Cohesive failures referred to fractures entirely within the dentin substrate or resin composite, reflecting failure of the bulk material rather than the interface. Mixed failure was characterized by a combination of adhesive and cohesive features, where both interfacial separation and material fractures were observed.

### 2.4. Field-Emission Scanning Electron Microscopy (FE-SEM) Analysis of the Bonded Interfaces

To elucidate the morphology of the resin–dentin interfaces, thirty-six additional third molars were prepared following the same protocols described for TBS testing. After composite build-up, each tooth was sectioned perpendicular to the bonded interface using a water-cooled diamond saw (IsoMet 5000) to produce slabs approximately 1.25 mm thick. Each slab was randomly divided into three groups, assigned to three artificial aging modes, and processed accordingly. The specimens were fixed in 2.5% glutaraldehyde for 12 h, rinsed with phosphate-buffered saline (PBS) and distilled water, dehydrated through a graded ethanol series, and dried with hexamethyldisilazane, following the method described by Perdigão et al. [34], and then examined using FE-SEM (Apreo S; Bruker, Billerica, MA, USA).

### 2.5. Statistical Analysis

An independent two-way analysis of variance (ANOVA) was conducted to assess the statistical significance of the μTBS results and elucidate the effects of aging conditions and adhesive systems. A post-hoc analysis was conducted using the Bonferroni test at a significance level of 0.05. Statistical analyses were performed using GraphPad Prism (version 10.4.0; GraphPad Software Inc., San Diego, CA, USA).

## 3. Results

### 3.1. μTBS Assessment

**Figure 2:**
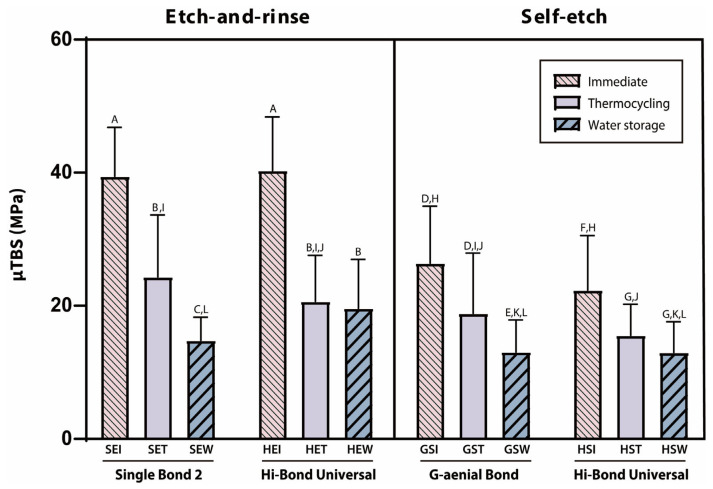
Mean micro-tensile bond strength (μTBS) values expressed in megapascals (MPa) for each group according to application modes and aging conditions. Specimens were grouped according to adhesive (Single Bond 2 [S], Hi-Bond Universal [H], G-ænial Bond [G]), application mode (etch-and-rinse [E] or self-etch [S]), and aging protocols (immediate [I], thermocycling [T], and water storage [W]). Groups sharing the same letter are not significantly different. Error bars indicate standard deviation.

The μTBS test results are shown in Figure 2. As illustrated in Figure 2, artificial aging reduced the μTBS of all adhesives tested, although the magnitude of reduction varied by adhesive and application mode.

Two-way ANOVA confirmed that both aging conditions and application mode exerted highly significant effects on μTBS (*p* < 0.0001). Correlation analysis revealed significant associations between μTBS and both aging conditions and application modes across all experimental variables (*p* < 0.0001; Table 2). Under immediate testing conditions (SEI, HEI, GSI, HSI), μTBS was consistently higher than after thermocycling or long-term water storage, irrespective of the adhesive employed. In the immediate condition, the etch-and-rinse modes (SEI and HEI) consistently produced significantly higher μTBS than the self-etch modes (GSI and HSI) (*p* < 0.0001). Both thermocycling and water storage produced a substantial decline in μTBS—ranging approximately 30–50%—thereby confirming that aging undermines bond durability (*p* < 0.0001). Notably, the water storage group for Hi-Bond Universal in the etch-and-rinse mode (HEW) demonstrated higher μTBS than Single Bond 2 with the same aging condition, whereas no significant differences were observed between the other paired groups (SET vs. HET, GST vs. HST, and GSW and HSW).

### 3.2. Failure Mode Analysis

**Figure 3:**
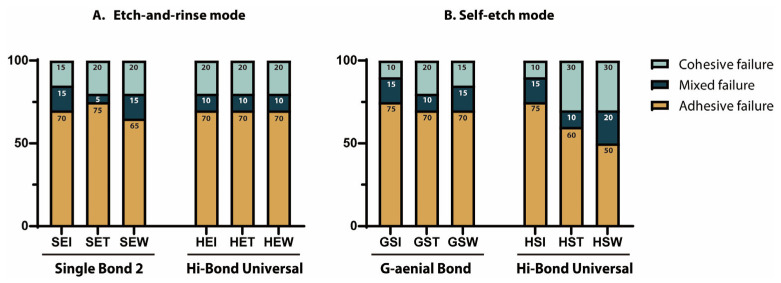
Percentage of each type of failure mode in each group according to aging conditions. Failures were classified as adhesive (failure at the adhesive–dentin interface), mixed (combination of adhesive and cohesive failures), or cohesive (failure within the dentin or composite). Groups were arranged according to adhesive (Single Bond 2 [S], Hi-Bond Universal [H], G-ænial Bond [G]), application mode (etch-and-rinse [E] or self-etch [S]), and aging protocols (immediate [I], thermocycling [T], and water storage [W]). Values within the bars represent the percentage of failure modes for each group.

Figure 3 shows the failure mode distributions of all experimental groups. Under immediate testing conditions (SEI, HEI, GSI, and HSI), adhesive failures were predominant, regardless of the application mode. Cohesive failures within either the composite or dentin substrate and mixed failures were rare.

As specimens underwent thermocycling and water storage, the proportion of cohesive failures increased in HST and HSW groups, accompanied by a corresponding decline in adhesive failures. Under 6-month water-storage conditions, cohesive failures increased further, while mixed-mode fractures also became more frequent.

### 3.3. FE-SEM Analysis of the Bonded Interfaces

**Figure 4:**
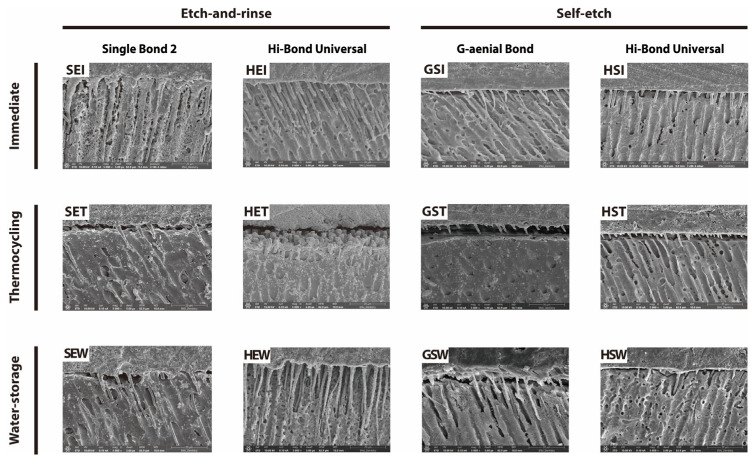
FE-SEM micrographs of the resin–dentin interfaces in intermediate, thermocycling, and water storage groups (magnification × 5000 scale bar = 20 μm). Adhesives were tested in etch-and-rinse or self-etch modes under different aging conditions (SEI, HEI, GSI, HSI, SET, HET, GST, HST, SEW, HEW, GSW, HSW).

The bonded interfaces for each experimental group are shown in Figure 4. In the etch-and-rinse mode, long resin tags and intact hybrid layers were observed in the immediate mode of Single Bond 2 and Hi-Bond Universal (SEI and HEI). After thermocycling, a detached hybrid layer was observed for both adhesives (SET and HET). Following water storage, Single Bond 2 also exhibited hybrid layer detachment, whereas Hi-Bond Universal retained an intact interface (SEW and HEW). In the self-etch mode, the immediate groups for G-ænial Bond and Hi-Bond Universal displayed short resin tags and relatively thick hybrid layers (GSI and HSI). However, after thermocycling or water storage, hybrid layer detachment was evident in both adhesives (GST, HST, GSW, and HSW).

## 4. Discussion

We aimed to evaluate the bond strength of Hi-Bond Universal, a novel MBG-containing universal adhesive, under different artificial aging protocols. These results led to the partial rejection of the null hypothesis because the μTBS of Hi-Bond Universal in etch-and-rinse mode was higher than that of Single Bond 2 after water storage.

Thermocycling and water storage were employed to simulate the intraoral environment. Thermocycling induces volumetric changes owing to differential thermal expansion between tooth structures and restorative materials, thereby weakening the adhesive interface. Numerous studies have recognized thermocycling as a reliable method to evaluate the effects of thermal stress on adhesive interfaces [3,4,35]. Water storage mimics prolonged moisture exposure in the oral cavity, activating endogenous matrix metalloproteinases (MMPs) and subsequently degrading collagen fibrils within the hybrid layer [11]. Within the hybrid layer, collagen fibrils are susceptible to enzymatic breakdown when exposed to prolonged moisture, as water storage can activate endogenous MMPs. This enzymatic activity gradually compromises the structural integrity of the adhesive interface, leading to a decrease in bond strength over time [36]. In this study, composite–dentin beams were produced and artificially aged for the rapid degradation of the adhesive interface. According to Gale and Darvell, the effect of 10,000 cycles of thermocycling was like that of 1 year of water storage. In the same way, 5000 thermocycling and 6-month water storage applied in this study can be considered to exert comparable degradative stress on the adhesive interface [37].

The μTBS values of the SEI and HEI were statistically comparable, indicating that incorporation of MBG into the universal adhesive did not compromise the initial bond strength. This finding is consistent with previous studies reporting negligible differences in immediate bond strength when MBG was incorporated into adhesive systems [38,39,40]. After thermocycling, the μTBS of HET and HST did not significantly differ from those of the SET and GST groups, suggesting that the mechanical stress induced by thermocycling limited the beneficial effects of MBG-mediated MMP inhibition. However, the μTBS of HEW remained significantly higher than that of SEW, demonstrating a protective effect of MBG under hydrolytic conditions.

According to the manufacturer’s explanation, the MBG incorporated into Hi-Bond Universal is silanized and well-dispersed. Non-silanized inorganic components can negatively influence the mechanical properties of composite materials. Previous research has shown that silanized bioactive glass particles can act as an inorganic filler and increase the mechanical performance of composite resin [41]. In a similar way, MBG in Hi-Bond Universal does not negatively affect the mechanical properties of the adhesives. μTBS of Hi-Bond Universal after thermocycling was similar to that of conventional adhesives, regardless of the application modes. It means that MBG in the Hi-Bond Universal does not have a harmful effect on the mechanical properties.

The findings of this study also suggest that incorporating MBG into dental adhesives effectively inhibits MMP activation following etch-and-rinse application, potentially enhancing bond durability. This inference aligns well with previous studies. For example, Osorio et al. demonstrated that resin formulations containing bioactive glass strongly suppressed the enzymatic activity of MMPs, substantially reducing adhesive interface degradation over time [42]. MBG inhibits MMPs mainly through its ion release and mesoporous structure. The sustained release of calcium and phosphate ions promotes remineralization of demineralized dentin, thereby minimizing collagen exposure to enzymatic attack. Calcium ions may also interfere with MMP catalytic sites, while local alkalization from released ions creates conditions unfavorable for MMP activity. The mesoporous architecture supports prolonged ion delivery, further enhancing this inhibitory effect. Collectively, these mechanisms help preserve the hybrid layer and improve resin–dentin bond durability [43]. In contrast, the bioactive benefits of Hi-Bond Universal were attenuated in the self-etch mode. This technique produced a shallower demineralization depth and thinner hybrid layer, which limited both resin penetration and the direct contact between MBG particles and collagen fibrils [44]. FE-SEM analysis (Figure 4-GSI and HSI) clearly showed that in the self-etch groups, the smear layer hindered deeper resin infiltration into dentin, resulting in a comparatively superficial adhesive interface. Consequently, MBG’s ability to release calcium and phosphate ions directly at the adhesive–dentin junction was diminished, reducing its MMP inhibitory effect [45]. Mazzoni et al. demonstrated that MMP activity was approximately three times higher in the etch-and-rinse mode than in the self-etch mode, attributing this difference to the restricted interaction between bioactive components and exposed collagen fibrils caused by shallower demineralization and incomplete infiltration characteristics of the self-etch protocols [45]. Similarly, the self-etch mode of MBG-containing adhesives offered no appreciable long-term durability advantage compared with conventional adhesives, underscoring the importance of selecting an appropriate etching strategy to maximize MBG’s clinical.

In the immediate groups, adhesive failure was predominant in all groups, indicating that initial debonding occurred primarily at the resin–dentin interface. This observation aligns with the well-established understanding that the immediate bond strength is determined largely by the integrity of the hybrid layer and the degree of resin infiltration into the dentin substrate. However, after thermocycling and water storage, there was a marked increase in the incidence of cohesive and mixed failures. The higher frequency of cohesive failures suggests that aging compromised the structural integrity of the adhesive layer or the underlying dentin [3,11,46]. Additionally, the rise in mixed failures implies partial interfacial breakdown in combination with bulk material breakdown. Collectively, these findings indicate that artificial aging progressively undermines the stability of the adhesive interface, shifting the primary failure mode from the interface to the bulk of the adhesive or dentin, thereby reducing overall restoration durability.

FE-SEM provided corroborative morphological evidence for the bond strength results by revealing clear microstructural differences between adhesive protocols. In the immediate condition, specimens treated with the etch-and-rinse method exhibited long, uniformly distributed resin tags that penetrated deeply into the dentinal tubules (Figure 4-SEI and HEI), whereas self-etch specimens displayed shorter and more heterogeneous tag formation (Figure 4-GSI and HSI). Following thermocycling, all groups, regardless of the application mode, demonstrated partial detachment at the resin–dentin interface, which manifested as shortened resin tags and interfacial gaps (Figure 4-SET, HET, GST, and HST). By contrast, after water storage, HEW specimens exhibited the most extensive resin tags formation accompanied by mineral precipitates at the interface (Figure 4-HEW), which may indicate improved hydrolytic stability and MBG-mediated bioactive remineralization.

Clinically, these findings suggest that incorporating MBG into a universal adhesive system does not compromise immediate bond strength and may preserve long-term μTBS when applied in an etch-and-rinse mode. By selectively removing the smear layer and exposing collagen fibrils, the etch-and-rinse mode facilitates deeper resin infiltration and more intimate contact between MBG particles and the demineralized dentin matrix [47]. This enhanced interfacial interaction inhibits endogenous MMP activity, thereby reducing collagen degradation and maintaining a statistically significant bond strength advantage over MBG-free adhesives during prolonged intraoral service [43].

This study has limitations that affect the extrapolation of the findings to clinical scenarios. Although thermocycling and water storage are widely used artificial aging methods, they do not fully reproduce the multifactorial challenges of the oral cavity. For example, masticatory function introduces cyclic mechanical loading and fatigue stress on adhesive interfaces, which can cause progressive degradation of bond strength and interfacial stability beyond what static aging protocols simulate [43]. The oral complex microbiota also produces acids and proteolytic enzymes within biofilms, chemically challenging the adhesive–resin interface in ways not modeled by conventional aging methods [48]. Furthermore, thermocycling parameters such as temperature range and dwell time vary between studies and may inadequately mimic natural intraoral thermal fluctuations and associated stresses [49]. In addition, diet- and saliva-related pH variations accelerate adhesive degradation but are not captured by simple water storage models. Finally, the aging duration employed in this study, while extended, remains limited compared with the actual clinical service life of restorations exposed to combined mechanical, biological, and chemical challenges over many years. Therefore, long-term in vivo studies are needed to corroborate these in vitro findings under realistic oral conditions.

## 5. Conclusions

In conclusion, this study demonstrated that MBG-containing universal adhesives applied in the etch-and-rinse mode maintained a significantly higher bond strength after water storage compared with conventional adhesives, whereas no difference was observed after thermocycling. By contrast, when applied in the self-etch mode, no significant differences in bond strength or morphology were detected.

These outcomes are likely attributable to MBG’s ability to inhibit MMP activity at the adhesive–dentin interface. These results indicate that in clinical practice, MBG-containing adhesives in the etch-and-rinse mode should be used to maximize their bioactive benefits.

## Figures and Tables

**Table 1:** Compositions of materials used in this study.

Material	Main Composition	Manufacturer
Single Bond 2	Ethanol, bis-GMA, silane-treated silica particles, HEMA, glycerol, dimethacrylate, water, copolymer of acrylic and itaconic acids, UDMA, diphenyliodonium hexafluorophosphate, EDMAB	3M ESPE, St. Paul, MN, USA
G-ænial Bond	UDMA, bis-MEPP, TEGDMA, silicon dioxide, strontium glass, photo-initiator, pigment	GC, Tokyo, Japan
Hi-Bond Universal	Mesoporous bioactive glass, 10-MDP, bis-GMA, HEMA, water, ethanol, silane coupling agent, photo-initiator, accelerators, etc.	MEDICLUS, Cheongju, Republic of Korea
Any-Com	Bis-GMA, UDMA, TEGDMA, barium glass, silicon dioxide, pho-to-initiators, accelerators, other additives	MEDICLUS, Cheongju, Republic of Korea
Artificial saliva	NaCl, 0.4 g; KCl, 0.4 g; CaCl_2_•2H_2_O, 0.795 g; NaH_2_PO_4_•2H_2_O, 0.78 g; Na_2_S•9H_2_O, 0.005 g; NH_2_CONH_2_, 1.0 g; distilled water, 1000 mL (pH 6.7)	Biosesang, Seongnam, Republic of Korea

**Table 2:** Results of two-way ANOVA test.

	F Value	*p* Value
Aging	51.85	<0.0001
Application mode	86.29	<0.0001
Aging × Application mode	7.245	<0.0001

Two-way ANOVA results showing F values and corresponding significance levels (*p* values) for aging, application mode, and their interaction.

## Data Availability

The original contributions presented in this study are included in the article. Further inquiries can be directed to the corresponding author.

## Abbreviations

The following abbreviations are used in this manuscript:

ANOVA	Analysis of variance
BAG	Bioactive glass
Bis-GMA	Bisphenol A-glycidyl methacrylate
Bis-MEPP	Bisphenol A-ethoxylate dimethacrylate
E	Etch-and-rinse
EDMAB	Ethyl-4-(dimethylamino)benzoate
FE-SEM	Field-emission scanning electron microscopy
G	G-ænial Bond
H	Hi-Bond Universal
HEMA	2-hydroxyethyl methacrylate
I	Immediate
LED	Light-emitting diode
MBG	Mesoporous bioactive glass
MDP	Methacryloyloxydecyl dihydrogen phosphate
MMP	Matrix metalloproteinase
PBS	Phosphate-buffered saline
S	Single Bond 2 (first component of the abbreviation) Self-etch (second component of the abbreviation)
T	Thermocycling
TEGDMA	Triethylene glycol dimethacrylate
UDMA	Urethane dimethacrylate
W	Water storage
μTBS	Micro-ternsile bond strength
